# Corn Meal

**Published:** 1869-02

**Authors:** 


*Corn Meal.*—We thought we knew something about this article of diet, having been largely indebted to it for our juvenile nutriment, and having a very good appetite for it at this late day. We are startled however, by some discoveries which the distinguished English lecturer, Dr. Letheby, has made in reference to its physiological or rather pathological nature.

Dr. Letheby, M. A., M.B., &c., enlightens his English audience after the following fashion:

"*Maize* or *Indian Corn* is one of the most extensivly used grains in the world. It enters largely into the food of the inhabitants of America, Italy, Corsica, Spain, the South of France and the Danish Principalities. Since the famine in Ireland, it has there also become a common article of diet, especially when potatoes are dear; but its flavor is harsh and peculiar, and nothing but a scarcity of more agreeable food reconciles people to its use. The *young grain* called *cob !!* is however more palatable, and forms when *boiled in milk*, an American luxury, which *takes the place of green peas!!!*"

The grain is said to cause disease when eaten for a long time and without other meal, the symptoms being a scaly eruption upon the hands, great prostration of the vital powers and death after a year or so, with extreme emaciation

We never were so startled but once before, and that was on reading a formidable array of symptoms described by a homœopathist as following the administration of one of his drugs. Among these were profound dejection and despair of God's mercy. The medicine which produced these pernicious effects turned out in the end to be only our ancient and innocent friend, common salt. We can imagine the smile which will pass over the faces of our readers, as they peruse Dr. Letheby's array of symptoms, and remember the sleek greasy negroes, and the round faced, rosy children who used to eat corn bread from the first of January to the thirty-first of December, as their standard farinaceous food. The information is paralelled as to value and accuracy only by that luminous account of corn cobs boiled in milk and used as a substitute for green peas, for which one American at least returns his thanks as for an entirely new idea.

